# Symptoms of Mental Health Conditions and Suicidal Ideation Among State, Tribal, Local, and Territorial Public Health Workers — United States, March 14–25, 2022

**DOI:** 10.15585/mmwr.mm7129a4

**Published:** 2022-07-22

**Authors:** Ahoua Koné, Libby Horter, Isabel Thomas, Ramona Byrkit, Barbara Lopes-Cardozo, Carol Y. Rao, Charles Rose

**Affiliations:** 1 CDC COVID-19 Emergency Response Team.

An increase in adverse mental health symptoms occurred in the general population at the onset of the COVID-19 pandemic, which peaked in 2020 and subsequently decreased (*1*–*3*). The pandemic exacerbated existing stress and fatigue among public health workers responding to the public health crisis.* During March–April 2021, a survey of state, tribal, local, and territorial (STLT) public health workers found that 52.8% of respondents experienced symptoms of at least one of the following mental health conditions: depression, anxiety, or posttraumatic stress disorder (PTSD) (*4*); however, more recent estimates of mental health symptoms among this population are limited. To evaluate trends in these conditions from the previous year, the prevalence of symptoms of mental health conditions and suicidal ideation, a convenience sample of STLT public health workers was surveyed during March 14–25, 2022. In total, 26,069 STLT public health workers responded to the survey. Among respondents,^†^ 6,090 (27.7%) reported symptoms of depression, 6,467 (27.9%) anxiety, 6,324 (28.4%) PTSD, and 1,853 (8.1%) suicidal ideation. Although the prevalences of depression, anxiety, and PTSD among public health workers were lower (p<0.001)^§^ among 2022 survey respondents compared with those of 2021 survey respondents (*4*), the prevalences of symptoms of suicidal ideation, anxiety, depression, and PTSD remained high among those who worked >60 hours per week (range = 11.3%–45.9%) and those who spent ≥76% of their work time on COVID-19 response activities (range = 9.0%–37.6%). Respondents were less likely to report mental health symptoms if they could take time off (prevalence ratio [PR] range = 0.48–0.55), or if they perceived an increase in mental health resources from their employer (PR range = 0.58–0.84). To support the mental health of public health workers, public health agencies can modify work-related factors, including making organizational changes for emergency responses and facilitating access to mental health resources and services.^¶^

During March 14–25, 2022, a nonprobability-based, self-administered, anonymous, web-based survey was disseminated to a convenience sample of public health workers who worked in U.S. STLT health departments for at least part of 2021.** The electronic survey link was distributed via email to national public health membership organizations, which shared the link with approximately 27,000 members with the request that members in a supervisory role cascade the survey to all public health workers within their respective organizations.^††^ The survey included questions on demographic characteristics, work history, traumatic events or stressors experienced since March 2021, employer-provided resources, and self-reported mental health symptoms of anxiety, depression, PTSD, or suicidal ideation within the previous 2 weeks. A similar convenience sample approach, survey instrument, and methodology were used in March 2021 (*4*). Mental health conditions were defined using validated instruments to evaluate symptoms of anxiety (2-item General Anxiety Disorder [GAD-2] questionnaire), depression (9-item Patient Health Questionnaire [PHQ-9]), and PTSD (6-item Impact of Event Scale [IES-6])^§§^ (*4*). One item from PHQ-9 was used to evaluate suicidal ideation.^¶¶^ Prevalences of depression, anxiety, PTSD, and suicidal ideation were stratified by demographic characteristics, workplace factors, stressors experienced, and coping mechanisms. Bivariate PRs of the four mental health conditions were calculated separately using Poisson regression with 95% CIs. Response frequencies from the 2021 and the 2022 surveys were tabulated, and prevalences (percentages) and 95% CIs of mental health outcomes were compared. Analyses were conducted using SAS (version 9.4; SAS Institute); p<0.05 or CIs for the PR that exclude 1.0 were considered statistically significant. This activity was reviewed by CDC and conducted consistent with applicable federal law and CDC policy.***

Overall, approximately one half of respondents (48.0% [95% CI = 47.3%–48.7%]) (A Koné, CDC, unpublished data, 2022) experienced symptoms of at least one of the mental health conditions of depression, anxiety, or PTSD.^†††^ The most commonly reported mental health condition was PTSD (28.4%) followed by anxiety (27.9%), depression (27.7%), and suicidal ideation (8.1%) (Table 1). The prevalences of depression, anxiety, and PTSD among public health workers were lower (−3.1%, −2.4%, and −8.4%, respectively) (p<0.001) among 2022 survey respondents compared with 2021 survey respondents (*4*). Respondents who identified as multiple races reported the highest prevalences of symptoms of depression (31.4%), anxiety (33.5%), and PTSD (34.4%) compared with other races. Most (91.4%) respondents worked ≥1 year in public health. Respondents who had spent ≥76% of work time on COVID-19 response activities were more likely to experience depression (PR = 1.38), anxiety (PR = 1.35), and PTSD (PR = 2.43), compared with public health workers not working on COVID-19. Respondents who worked >60 hours per week were more likely than were respondents working ≤40 hours per week to experience depression (PR = 1.73), anxiety (PR = 1.48), PTSD (PR = 2.07), and suicidal ideation (PR = 1.50). The percentage of symptoms of mental health conditions and suicidal ideation increased with the percentage of time working on COVID-19 response activities, especially among those who spent ≥76% of their work time on COVID-19 (range = 9.0%–37.6%) and for those who worked >60 hours per week (range = 11.3%–45.9%). This difference was most notable for PTSD in both 2021 and 2022 (Table 2). In 2021, among public health workers who had spent ≥76% of work time on COVID-19 response activities and worked ≤40, 41–60, and >60 hours per week, the prevalences of PTSD were 35.8%, 47.3%, and 58.7%, respectively, representing increases of 70.5%, 82.6%, and 109.6%, respectively, over those among public health workers not working on COVID-19. In addition, compared with 2021, the PRs for PTSD increased in 2022 for respondents who worked >60 hours per week and spent any time on COVID-19 activities: among those who spent 1%–25%, 26%–50%, 51%–75%, and ≥76% of time on COVID-19 activities, PTSD PRs during 2021 and 2022 were 1.14 and 1.39, 1.02 and 1.67, 1.67 and 2.19, and 2.10 and 2.48, respectively.

**TABLE 1:** Symptoms of depression, anxiety, posttraumatic stress disorder, and suicidal ideation among state, tribal, local, and territorial public health workers (N = 26,069) during the preceding 2 weeks, by demographic characteristics — United States, March 14–25, 2022

Characteristic	No. (%)	Depression* (n = 21,965)^†^		Anxiety* (n = 23,176)^†^		PTSD* (n = 22,261)^†^		Suicidal ideation (n = 22,862)^†^	
		%	PR (95% CI)	%	PR (95% CI)	%	PR (95% CI)	%	PR (95% CI)
**Overall**	26,069*	**27.7**	**(27.1–28.3)**	**27.9**	**(27.3–28.5)**	**28.4**	**(27.8–29.0)**	**8.1**	**(7.8–8.5)**
**Jurisdiction type**									
Local	13,383 (51.3)	27.1	0.95 (0.91–1.00)	27.8	0.99 (0.95–1.03)	29.7	1.11 (1.06–1.15)	7.8	0.93 (0.85–1.01)
Tribal	340 (1.3)	29.0	1.02 (0.85–1.23)	26.3	0.93 (0.77–1.13)	31.1	1.16 (0.97–1.38)	7.5	0.89 (0.59–1.34)
Territorial	104 (0.4)	23.3	0.82 (0.56–1.20)	24.1	0.86 (0.59–1.25)	27.4	1.02 (0.72–1.44)	9.1	1.08 (0.56–2.09)
State	12,242 (47.0)	28.4	Ref	28.1	Ref	26.9	Ref	8.4	Ref
**Age group, yrs**									
≤29	3,235 (15.4)	34.8	2.10 (1.90–2.32)	41.2	2.91 (2.63–3.23)	33.8	1.80 (1.64–1.97)	13.5	3.17 (2.60–3.87)
30–39	5,124 (24.5)	31.4	1.90 (1.72–2.09)	34.2	2.41 (2.18–2.67)	33.3	1.77 (1.62–1.94)	8.9	2.10 (1.72–2.56)
40–49	4,893 (23.3)	28.9	1.75 (1.58–1.92)	27.8	1.97 (1.77–2.18)	30.0	1.60 (1.46–1.75)	8.3	1.95 (1.59–2.38)
50–59	4,942 (23.6)	25.4	1.53 (1.39–1.69)	21.9	1.55 (1.39–1.72)	25.1	1.33 (1.21–1.47)	6.3	1.47 (1.20–1.82)
≥60	2,763 (13.2)	16.5	Ref	14.2	Ref	18.8	Ref	4.3	Ref
**Gender**									
Female	19,397 (82.6)	27.8	1.10 (1.03–1.17)	28.2	1.18 (1.11–1.25)	28.5	1.09 (1.02–1.15)	7.2	0.67 (0.60–0.74)
Transgender or nonbinary	220 (0.9)	55.7	2.20 (1.92–2.52)	52.5	2.19 (1.91–2.51)	51.9	1.98 (1.72–2.28)	31.7	2.92 (2.34–3.64)
Male	3,853 (16.4)	25.3	Ref	24.0	Ref	26.3	Ref	10.9	Ref
**Race or ethnicity**									
Hispanic	2,609 (11.6)	27.8	0.98 (0.91–1.05)	26.6	0.93 (0.87–1.00)	32.1	1.16 (1.09–1.23)	8.8	1.13 (0.98–1.30)
AI/AN, non-Hispanic	184 (0.8)	30.5	1.07 (0.86–1.35)	26.6	0.93 (0.73–1.19)	32.6	1.17 (0.94–1.46)	8.4	1.08 (0.66–1.75)
Asian, non-Hispanic	1,237 (5.5)	25.5	0.90 (0.81–1.00)	27.6	0.97 (0.88–1.06)	29.4	1.06 (0.96–1.16)	10.7	1.37 (1.15–1.63)
Black, non-Hispanic	1,985 (8.8)	20.5	0.72 (0.66–0.80)	20.9	0.73 (0.67–0.80)	23.8	0.86 (0.78–0.93)	5.5	0.71 (0.58–0.86)
NH/OPI, non-Hispanic	132 (0.6)	27.6	0.98 (0.73–1.30)	22.3	0.78 (0.57–1.08)	32.3	1.16 (0.90–1.50)	12.6	1.62 (1.02–2.57)
Multiple races, non-Hispanic	590 (2.6)	31.4	1.11 (0.97–1.26)	33.5	1.17 (1.04–1.32)	34.4	1.24 (1.10–1.39)	12.3	1.58 (1.26–1.98)
White, non-Hispanic	15,765 (70.1)	28.3	Ref	28.5	Ref	27.8	Ref	7.8	Ref
**Highest educational degree attained**									
Bachelor’s	8,967 (38.2)	28.3	1.00 (0.94–1.05)	28.6	1.10 (1.04–1.16)	27.4	1.14 (1.07–1.21)	8.6	1.19 (1.06–1.34)
Graduate	9,093 (38.8)	26.5	0.93 (0.88–0.99)	28.1	1.08 (1.02–1.14)	31.9	1.33 (1.25–1.41)	8.1	1.12 (0.99–1.26)
Less than bachelor’s	5,387 (23.0)	28.4	Ref	26.0	Ref	24.0	Ref	7.2	Ref
**Hrs worked per wk**									
41–60	10,367 (43.2)	30.7	1.29 (1.24–1.35)	30.4	1.23 (1.17–1.28)	33.5	1.51 (1.45–1.58)	8.4	1.13 (1.03–1.23)
>60	1,350 (5.6)	41.2	1.73 (1.61–1.87)	36.8	1.48 (1.37–1.60)	45.9	2.07 (1.93–2.22)	11.3	1.50 (1.27–1.77)
≤40	12,277 (51.2)	23.8	Ref	24.8	Ref	22.2	Ref	7.5	Ref
**% Time spent on COVID-19 response activities**									
1–25	5,792 (24.4)	25.0	1.11 (1.02–1.22)	25.3	1.10 (1.01–1.20)	19.8	1.28 (1.14–1.43)	8.0	1.37 (1.15–1.63)
26–50	3,343 (14.1)	27.0	1.20 (1.09–1.33)	26.7	1.16 (1.06–1.28)	25.5	1.65 (1.47–1.85)	7.1	1.56 (1.39–1.75)
51–75	3,016 (12.7)	27.6	1.23 (1.11–1.36)	28.7	1.25 (1.14–1.37)	30.7	1.98 (1.77–2.21)	7.2	1.41 (1.23–1.61)
≥76	9,161 (38.6)	31.1	1.38 (1.27–1.51)	30.9	1.35 (1.24–1.46)	37.6	2.43 (2.20–2.69)	9.0	1.16 (0.99–1.37)
0	2,445 (10.3)	22.4	Ref	23.0	Ref	15.5	Ref	7.8	Ref
**Yrs worked in public health**									
<1	2,106 (8.6)	26.0	1.08 (0.99–1.18)	28.3	1.28 (1.18–1.40)	23.2	0.90 (0.82–0.99)	8.5	1.37 (1.15–1.63)
1–4	7,846 (32.1)	30.3	1.26 (1.19–1.33)	32.0	1.45 (1.37–1.53)	30.2	1.17 (1.10–1.23)	9.7	1.56 (1.39–1.75)
5–9	4,676 (19.1)	29.9	1.24 (1.17–1.33)	30.5	1.38 (1.30–1.47)	30.7	1.19 (1.12–1.26)	8.7	1.41 (1.23–1.61)
10–14	2,905 (11.9)	27.7	1.15 (1.07–1.24)	27.0	1.22 (1.13–1.32)	29.8	1.15 (1.07–1.24)	7.2	1.16 (0.99–1.37)
≥15	6,921 (28.3)	24.1	Ref	22.1	Ref	25.9	Ref	6.2	Ref
**Remember completing 2021 survey**									
Yes	7,527 (28.9)	28.5	1.04 (0.99–1.09)	28.4	1.03 (0.98–1.07)	31.3	1.15 (1.10–1.21)	8.4	1.06 (0.96–1.16)
No	18,529 (71.1)	27.4	Ref	27.7	Ref	27.1	Ref	8.0	Ref

**Abbreviations: **AI/AN = American Indian or Alaska Native; GAD-2 = 2-item General Anxiety Disorder; IES-6 = 6-item Impact of Event Scale; NH/OPI = Native Hawaiian or other Pacific Islander; PHQ-9 = 9-item Patient Health Questionnaire; PTSD = posttraumatic stress disorder; PR = prevalence ratio; Ref = referent group.

* Some categories might not sum to total number of respondents (26,069) because of missing data. Counts for each category are those who answered all validated survey questions for that symptom.

^†^ Respondents who scored ≥10.0 out of 27 on the PHQ-9 were categorized as being symptomatic for depression; those who scored ≥3.0 out of 6 on the GAD-2 were categorized as being symptomatic for anxiety; and respondents who scored ≥1.75 out of 4 on IES-6 were categorized as being symptomatic for PTSD. Respondents who indicated that they would be better off dead or thought of hurting themselves at any time in the past 2 weeks on the PHQ-9 were categorized as being symptomatic for suicidal ideation.

**TABLE 2:** Symptoms of posttraumatic stress disorder among state, tribal, local, and territorial public health workers, by percentage of work time spent on COVID-19 response activities and hours worked in a week — United States, March–April 2021 and March 14–25, 2022

No. of hrs worked per wk	% Time on COVID-19 response	2021 survey (Mar–Apr 2021) (N = 26,174)		2022 survey (Mar 14–25, 2022) (N = 26,069)	
		PTSD* prevalence (%)	PR (95% CI)	PTSD* prevalence (%)	PR (95% CI)
**≤40**	0	21.0	Ref	15.3	Ref
	1–25	21.4	1.02 (0.89–1.16)	17.8	1.16 (1.01–1.32)
	26–50	28.3	1.35 (1.17–1.55)	22.2	1.45 (1.25–1.68)
	51–75	31.1	1.48 (1.28–1.70)	24.3	1.58 (1.36–1.84)
	≥76	35.8	1.70 (1.50–1.92)	29.4	1.92 (1.70–2.17)
**41–60**	0	25.9	Ref	15.8	Ref
	1–25	28.7	1.11 (0.92–1.33)	23.2	1.47 (1.19–1.82)
	26–50	35.1	1.35 (1.13–1.63)	28.7	1.82 (1.47–2.25)
	51–75	39.0	1.50 (1.25–1.80)	34.0	2.16 (1.75–2.66)
	≥76	47.3	1.83 (1.54–2.17)	41.5	2.63 (2.15–3.22)
**>60**	0	28.0	Ref	20.0	Ref
	1–25	31.9	1.14 (0.57–2.28)	27.8	1.39 (0.62–3.11)
	26–50	28.7	1.02 (0.52–2.00)	33.3	1.67 (0.76–3.66)
	51–75	46.7	1.67 (0.88–3.16)	43.8	2.19 (1.05–4.57)
	≥76	58.7	2.10 (1.12–3.94)	49.5	2.48 (1.21–5.08)

**Abbreviations**: IES-6 = 6-item Impact of Event Scale; PTSD = posttraumatic stress disorder; PR = prevalence ratio; Ref = referent group.

* Self-reported symptoms of PTSD were evaluated; respondents who scored ≥1.75 out of 4 on the IES-6 were considered to be symptomatic for PTSD.

Since March 2022, respondents who reported feeling overwhelmed by workload or family and work balance were 2.35, 2.67, 2.90, and 2.98 times as likely to report symptoms of suicidal ideation, anxiety, depression, and PTSD, respectively, as were those not reporting feeling overwhelmed (Table 3). Public health workers who received job-related threats or felt bullied, threatened, or harassed because of their job reported the highest prevalences of PTSD (53.3% and 47.7%, respectively). Approximately one quarter of respondents (27.8%) who have left or were considering leaving public health were approximately twice as likely to report suicidal ideation (PR = 2.34) compared with those staying in the field. In addition, 73.9% of public health workers knew colleagues who left or were considering leaving public health. A total of 16,462 (75.4%) respondents were able to take time off from work. Public health workers who could take time off from work were less likely to report symptoms of depression (PR = 0.50), anxiety (PR = 0.55), PTSD (PR = 0.51), or suicidal ideation (PR = 0.48) compared with those unable to take time off. According to 75.5% of public health workers, their employer had not increased support for staff members’ mental health since March 2021. Respondents who reported an increase in mental health resources were less likely than were those who did not to report symptoms of depression (PR = 0.68), anxiety (PR = 0.71), PTSD (PR = 0.84), and suicidal ideation (PR = 0.58). Among public health workers who did perceive an increase in mental health resources, those considered to be most useful were demonstrating appreciation for staff members’ work (63.4%), telework options (58.2%), and flexible work schedules (55.0%) (A Koné, CDC, unpublished data, 2022).

**TABLE 3:** Symptoms of depression, anxiety, posttraumatic stress disorder, and suicidal ideation among state, tribal, local, and territorial public health workers (N = 26,069) during the past 2 weeks, by work factors — United States, March 14–25, 2022

Work factor	No. (%)	Depression* (n = 21,965)^†^		Anxiety* (n = 23,176)^†^		PTSD* (n = 22,261)^†^		Suicidal ideation (n = 22,862)^†^	
		%	PR (95% CI)	%	PR (95% CI)	%	PR (95% CI)	%	PR (95% CI)
**Overwhelmed by workload or family and work balance**									
Yes	14,916 (65.8)	35.8	2.90 (2.72–3.10)	35.4	2.67 (2.51–2.83)	36.7	2.98 (2.80–3.18)	10.1	2.35 (2.09–2.64)
No	7,738 (34.2)	12.3	Ref	13.3	Ref	12.3	Ref	4.3	Ref
**Disconnected from family and friends because of workload**									
Yes	11,310 (50.0)	40.1	2.61 (2.48–2.75)	39.4	2.43 (2.32–2.55)	41.5	2.74 (2.61–2.88)	11.7	2.59 (2.34–2.86)
No	11,309 (50.0)	15.4	Ref	16.2	Ref	15.2	Ref	4.5	Ref
**Inadequately compensated for work**									
Yes	14,120 (62.9)	34.0	1.99 (1.88–2.10)	33.7	1.89 (1.79–1.99)	34.9	2.02 (1.92–2.13)	9.9	1.92 (1.73–2.14)
No	8,325 (37.1)	17.1	Ref	17.8	Ref	17.3	Ref	5.1	Ref
**Unappreciated at work**									
Yes	12,045 (53.5)	36.9	2.12 (2.02–2.23)	36.4	2.02 (1.92–2.11)	37.1	2.01 (1.91–2.10)	11.0	2.28 (2.06–2.52)
No	10,485 (46.5)	17.4	Ref	18.1	Ref	18.5	Ref	4.8	Ref
**Experienced stigma or discrimination because of work**									
Yes	6,420 (28.5)	41.1	1.83 (1.75–1.91)	39.6	1.71 (1.64–1.78)	45.5	2.12 (2.04–2.21)	11.7	1.77 (1.62–1.94)
No	16,136 (71.5)	22.4	Ref	23.2	Ref	21.4	Ref	6.6	Ref
**Received job-related threats because of work**									
Yes	2,523 (11.2)	43.8	1.71 (1.62–1.80)	43.4	1.68 (1.60–1.77)	53.3	2.12 (2.03–2.21)	14.8	2.05 (1.84–2.29)
No	20,071 (88.8)	25.6	Ref	25.9	Ref	25.2	Ref	7.2	Ref
**Bullied, threatened, or harassed because of work**									
Yes	5,199 (23.0)	42.3	1.81 (1.74–1.89)	41.4	1.74 (1.67–1.82)	47.7	2.12 (2.04–2.21)	13.0	1.97 (1.80–2.16)
No	17,369 (77.0)	23.3	Ref	23.8	Ref	22.5	Ref	6.6	Ref
**Can take time off from work**									
Yes	16,462 (75.4)	22.3	0.50 (0.48–0.53)	23.1	0.55 (0.53–0.57)	23.1	0.51 (0.49–0.53)	6.4	0.48 (0.44–0.52)
No	5,365 (24.6)	44.2	Ref	42.0	Ref	44.9	Ref	13.4	Ref
**Left or considering leaving job**									
Yes	6,525 (27.8)	42.3	1.92 (1.84–2.00)	41.3	1.83 (1.76–1.91)	41.9	1.80 (1.73–1.88)	13.8	2.34 (2.14–2.55)
No	16,917 (72.2)	22.0	Ref	22.5	Ref	23.2	Ref	5.9	Ref
**Know colleagues who left or considering leaving**									
Yes	17,622 (73.9)	31.4	1.78 (1.67–1.89)	30.8	1.55 (1.46–1.64)	32.5	1.85 (1.74–1.97)	8.9	1.55 (1.38–1.74)
No	6,215 (26.1)	17.6	Ref	19.9	Ref	17.5	Ref	5.8	Ref
**Employer increased their support or resources for staff members’ mental health**									
Yes	5,412 (24.5)	20.7	0.68 (0.64–0.72)	21.5	0.71 (0.67–0.75)	24.9	0.84 (0.80–0.88))	5.3	0.58 (0.52–0.66)
No	16,712 (75.5)	30.4	Ref	30.2	Ref	29.7	Ref	9.1	Ref

**Abbreviations:** GAD-2 = 2-item General Anxiety Disorder; IES-6 = 6-item Impact of Event Scale; PHQ-9 = 9-item Patient Health Questionnaire; PTSD = posttraumatic stress disorder; PR = prevalence ratio; Ref = referent group.

* Some categories might not sum to total number of respondents (26,069) because of missing data. Counts for each category represent those who answered all validated survey questions for that symptom.

^†^ Respondents who scored ≥10.0 out of 27 on the PHQ-9 were categorized as being symptomatic for depression; those who scored ≥3.0 out of 6 on the GAD-2 were categorized as being symptomatic for anxiety; and respondents who scored ≥1.75 out of 4 on IES-6 were categorized as being symptomatic for PTSD. Respondents who indicated that they would be better off dead or thought of hurting themselves at any time in the past 2 weeks on the PHQ-9 were categorized as being symptomatic for suicidal ideation.

## Discussion

Public health workers who spent more time on COVID-19 response activities were more likely to report mental health symptoms, including PTSD. Compared with results of the 2021 survey of STLT public health workers (*4*), in 2022, prevalence of PTSD was 15.7% lower among public health workers who worked >60 hours per week and spent ≥76% on COVID-19. However, the PRs increased, and the prevalence of PTSD (49.5%) was higher for this group than the overall prevalence of PTSD (28.4%). Previous studies have documented that persons who work long hours are susceptible to experiencing negative mental health or physiologic outcomes (*5*,*6*).

Prolonged exposure to occupational stressors can lead to adverse mental health conditions and has been linked with high health care worker turnover during the COVID-19 pandemic (*7*,*8*). Respondents who left or were considering leaving public health were more likely to report symptoms of mental health conditions and suicidal ideation. Approximately three quarters of public health workers did not perceive an increase in employer-based mental health resources for staff members. According to the 2021 Public Health Workforce Interests and Needs Survey, public health workers were considering leaving their employment because of burnout, stress, and organizational culture (*9*). In addition, in the 2022 CDC survey of public health workers, respondents who expressed feeling bullied or threatened reported some of the highest prevalences of symptoms of mental health conditions and suicidal ideation. It is therefore important that public health agencies identify risk factors for workplace violence, recognize signs that public health workers are being bullied or threatened, and implement strategies to prevent and address these incidents.^§§§^

The findings in this report are subject to at least six limitations. First, the respondents were drawn from a nonprobability-based convenience sample of STLT public health workers who employed partial snowball sampling; thus, these findings are not generalizable to and might not represent the entire STLT public health workforce. Second, because of the survey distribution method and an approximation of the number of public health workers (range = 231,464–341,053) (*10*), a true response rate cannot be calculated. Third, although validated instruments were used to score respondents’ mental health symptoms, the score does not confirm a clinical diagnosis of a mental health disorder (*4*). Fourth, the data are subject to recall bias; some questions asked respondents to recall experiences since March 2021. Fifth, data came from cross-sectional surveys; therefore, the findings do not reflect changes in symptoms among the same persons over time. Finally, a multivariable analysis was not conducted, and it is possible that observed differences between surveys could be because of demographic or other variations between the two samples.

It is critical for public health agencies to invest in and develop their STLT public health workforce to address mental health, including symptoms of depression, anxiety, PTSD, and suicidal ideation. Investment in the current and future workforces might include training organizational leaders and supervisors to recognize, understand, and support staff members’ mental health needs. Organization-led initiatives, including reducing the number of hours or percentage of time public health workers work on an emergency response might also improve workforce health.

**Summary**

**What is already known about this topic?**

In 2021, state, tribal, local, and territorial (STLT) public health workers reported high levels of symptoms of at least one mental health condition (depression, anxiety, or posttraumatic stress disorder [PTSD]).

**What is added by this report?**

In a 2022 survey of 26,069 STLT public health workers, higher PTSD prevalence was associated with more weekly work hours and more time spent on COVID-19 response activities. Most (75.5%) respondents did not think their employer increased mental health support.

**What are the implications for public health practice?**

To support the mental health of public health workers, public health agencies can modify work-related factors, including making organizational changes for emergency responses and facilitating access to mental health resources and services.

## Footnotes

*
https://www.hhs.gov/about/news/2022/05/23/new-surgeon-general-advisory-sounds-alarm-on-health-worker-burnout-and-resignation.html

† Counts for mental health symptoms might not sum to total number of respondents (26,069) because of missing data. Counts for each category are those who answered all validated survey questions for that outcome: depression (21,965), anxiety (23,176), PTSD (22,261), and suicidal ideation (22,862).

§ Overall prevalence of symptoms of suicidal ideation was not statistically different from 2021 to 2022.

¶
https://www.cdc.gov/niosh/twh/guidelines.html

** Respondents who did not report working at an STLT public health agency or department for any amount of time in 2021 were excluded from the analysis.

†† Member associations and other organizations that participated were Association of Public Health Laboratories, Association of State and Territorial Health Officials, Council of State and Territorial Epidemiologists, National Association of County and City Health Officials, National Association of Community Health Workers, National Network of Public Health Institutes, and CDC Foundation.

§§ The PHQ-9 was used to score depression (score range = 0–27) and suicidal ideation (0–3), and respondents were considered symptomatic for depression if they scored ≥10. GAD-2 was used to score anxiety: each response option was assigned a value from 0 to 3, for a total range of 0–6, and respondents were considered symptomatic at a score of ≥3. To evaluate PTSD, the IES-6 was scored from 0 to 4 for each question for a total score range of 0–24; however, symptoms of PTSD were calculated as the mean of six questions. Respondents were considered symptomatic for PTSD if they scored ≥1.75.

¶¶ One item from PHQ-9, “How many days have you thought that you would be better off dead or thought of hurting yourself?” was used to evaluate suicide-related thoughts (referred to as suicidal ideation in the report).

*** 45 C.F.R. part 46, 21 C.F.R. part 56; 42 U.S.C. Sect. 241(d); 5 U.S.C. Sect. 552a; 44 U.S.C. Sect. 3501 et seq.

††† One item from PHQ-9, “How many days have you thought that you would be better off dead or thought of hurting yourself?” does not evaluate a condition; therefore, only reported symptoms of depression, anxiety, and PTSD were included in the calculation of respondents who reported at least one mental health condition.

§§§
https://www.osha.gov/workplace-violence
